# Hyaluronic acid production and characterization by novel *Bacillus subtilis* harboring truncated Hyaluronan Synthase

**DOI:** 10.1186/s13568-022-01429-3

**Published:** 2022-07-12

**Authors:** Fatemeh Sadat Amjad Zanjani, Shadi Afrasiabi, Dariush Norouzian, Gholamreza Ahmadian, Sara Ali Hosseinzadeh, Alireza Fayazi Barjin, Reza Ahangari Cohan, Malihe Keramati

**Affiliations:** 1 Department of Nanobiotechnology, New Technologies Research Group, Pasteur Institute of Iran, Tehran, Iran; 2 Department of Industrial and Environmental Biotechnology, National Institute for Genetic Engineering and Biotechnology (NIGEB), Tehran, Iran

**Keywords:** *Bacillus subtilis*, Hyaluronic acid, Hyaluronan synthase, Transmembrane domain, Truncation, Molecular weight

## Abstract

Hyaluronic Acid (HA) is a natural biopolymer that has important physiological and industrial applications due to its viscoelastic and hydrophilic characteristics. The responsible enzyme for HA production is Hyaluronan synthase (HAS). Although in vitro structure–function of intact HAS enzyme has been partly identified, there is no data on in vivo function of truncated HAS forms. In the current study, novel recombinant *Bacillus subtilis* strains harboring full length (RBSFA) and truncated forms of SeHAS (RBSTr4 and RBSTr3) were developed and HA production was studied in terms of titer, production rate and molecular weight (Mw). The maximum HA titer for RBSFA, RBSTr4 and RBSTr3 was 602 ± 16.6, 503 ± 19.4 and 728 ± 22.9 mg/L, respectively. Also, the HA production rate was 20.02, 15.90 and 24.42 mg/L.h^−1^, respectively. The findings revealed that RBSTr3 produced 121% and 137% more HA rather than RBSFA and RBSTr4, respectively. More interestingly, the HA Mw was about 60 kDa for all strains which is much smaller than those obtained in prior studies.

**Supplementary Information:**

The online version contains supplementary material available at 10.1186/s13568-022-01429-3.

## Key points

- The strains containing truncated forms of SeHAS enzysme are able to produce HA.
- The HA from all recombinant strains was the same and low Mw.
- Deletion of C-terminal region of SeHAS was not effective on Mw.

**Supplementary Information:**

The online version contains supplementary material available at 10.1186/s13568-022-01429-3.

## Introduction

Hyaluronic acid (HA) also known as hyaluronan, is a linear polysaccharide composed of disaccharide repeat units of GlcNAc (β1-3 D-N-acetyl glucosamine) and GlcA (β1-4 D-glucuronic acid) (Liu and Catchmark 2019). HA has important structural, physiological and biological functions due to its viscoelastic property and ability to keep a large capacity of water (Yoshimura et al. 2015). These properties have led to a wide range of clinical applications in pharmaceutical, biomedical and cosmetic industries (Westbrook et al. 2018a) including skin moisturizers, wound healing (Greenlee et al. 2017), visco-supplement injection, ophthalmic and abdominal surgeries (Westbrook et al. 2018a). Based on the source and processing means, the molecular weight (Mw) of HA varies over a wide range, from lower than 1000 daltons to several million daltons (Dovedytis et al. 2020). The biological effects of HA depend on the Mw as it regulates the physiological responses and receptor interactions (Liu et al. 2011; Waeijen-Smit et al. 2021).

The ordinary methods for HA commercial production are extraction from rooster combs and pathogenic *Streptococcus* fermentation. Neither is an ideal source due to safety concerns such as related avian allergens and exotoxin contaminations and also HA degradation during the purification process and hyaluronidase digestion. Therefore, it would be advantageous to develop alternative sources for HA production such as GRAS (Generally Recognized As Safe) strains including *Bacillus subtilis*, *Agrobacterium*, *Escherichia. coli* and *Lactococcus lactis* (Gunasekaran et al. 2020; Kogan et al. 2007; Sze et al. 2016). Among GRAS, *B. subtilis* strains have several advantages such as high yield and appropriate quality of the final product that proved them as a choice host for HA production (Manfrão‐Netto et al. 2022; Westbrook et al. 2018a; Westbrook et al. 2018b). Furthermore, *B. subtilis*-derived HA is exotoxin and endotoxin-free that secreted into the culture medium which simplifies the downstream process (Westbrook et al. 2016).

*B.subtilis* strains have the intrinsic biosynthesis pathways of GlcNAc and GlcA, thus the development of recombinant HA producer strains just requires the enzyme for binding and polymerization of those mono-saccharides. Some of these recombinant strains were developed by cloning of different *Streptococcus* HAS genes (Hyaluronan Synthase) (Agarwal et al. 2019; Rehm 2010; Yu and Stephanopoulos 2008). The two streptococcal HAS enzymes of *Streptococcus pyogenes* (SpHAS) and *Streptococcus equisimilis (*SeHAS) are the smallest members of the HAS family containing 419 and 417 amino acids, respectively with high production rate than that of the other HAS enzymes (Heldermon et al. 2001).

The spHAS is a membrane enzyme that structurally consists of four TMDs (Trans Membrane Domain) and two membrane-associated regions. It was speculated that the intracellular domain (located between the TMD2 and TMD 3) mediates substrate binding and catalytic activity (Aaltonen and Silow 2008; Heldermon et al. 2001; Yang et al. 2017).Although there is no topological data for SeHAS derived from *S. equisimilis,* prior structure–function studies via amino acid substitutions or deletions showed that he biological activity of SeHAS in terms of production rate and HA Mw depends on enzyme structure (Baggenstoss et al. 2017; Yang et al. 2017).

To the best of our knowledge, there are no reports on the effects of TMD deletion on titer, production rate and Mw of HA in recombinant *B. subtilis* platform. This lack of investigation becomes ever more highlighted when comparing the highly inconsistent results among the various in vitro studies (Baggenstoss et al. 2017; Yang et al. 2017). In the current study, novel *B. subtilis* hosts harboring full and truncated forms of SeHAS were developed and investigated for HA production. Furthermore, the Mw of HA products during batch fermentation by different hosts was compared for a comprehensive conclusion on the role of TMD of SeHAS on product properties.

## Material and methods

### Bacterial strains, plasmids and culture conditions

The bacterial strains and plasmids used and developed in this study were summarized in Table 1. The *B. subtilis* strain 168 was purchased from *Bacillus* Genetic Stock Centre (BGCS) and *E. coli* TOP10 cells were obtained from Invitrogen Co. The pDG148 vector was a gift from Ezio Riccac (Federico II University of Naples, Italy). All strains were cultured in LB medium containing appropriate antibiotics at 37 °C, 200 rpm. Ampicillin (100 μg/mL) and kanamycin (20 μg/mL) were used for plasmid selection in *E. coli* and *B. subtilis*, respectively. The pDG148 shuttle expression vector was used for the construction of expression enzyme vectors. For protein expression under the control of *lac* promoter, the bacterial culture was induced by IPTG at a final concentration of 0.6 mM.

**Table 1:** Bacterial Strains and Plasmids were used or developed in this study

Strain/Plasmid	Description	Reference
Strains		
* E. coli* TOP10	Fˊ(*lacIq* Tn10 (*tetR*)) *mcrA*D Δ*lacX74 deoR nupG recA1 araD139*Δ(*ara-leu*)7697 *galU galK rpsL* (*strR*) *endA1*λ- cloning host	Invitrogen
* B. subtilis* 168	trpC2 (ATCC 33,712, (ATCC, Manassas, VA, USA)	(Harwood 1992)
RBSFA	*B. subtilis* 168 carrying pDG148-hasA	Developed in this study
RBSTr3	*B. subtilis* 168 carrying pDG148Tr3	Developed in this study
RBSTr4	*B. subtilis* 168 carrying pDG148Tr4	Developed in this study
Plasmids		
pDG148	Multicopy *B. subtilis* and *E. coli shuttle* vector; Ap^r^, Km^r^, replicative	(Stragier et al. 1988)
pDG148-hasA	pDG148 carrying *hasA* gene	Developed in this study
pDG148-Tr3	pDG148 carrying *Tr3* gene^*^	Developed in this study
pDG148-Tr4	pDG148 carrying *Tr4* gene^*^	Developed in this study

^*^Tr is truncated gene/protein

### Design and construction of full-length and truncated forms of *hasA* gene

In the case of full-length construct, the coding sequence of *hasA* gene (1251 bp) of *S. equisimilis* (Gene ID: 2,655,099) was optimized and designed for expression in *B. subtilis* 168. Two stop codons and a 6-HisTag at the C-terminus and a terminator sequence containing 82 bp for enhancement of mRNA stability were included just after 6-His tag region within the expression construct. The nucleotide sequence and schematic representation of full-length construct was shown in Additional file 1: Fig. S1. The nucleotide and amino acid sequence of optimized full length and its truncated forms including Tr4 (1128 bp, 376 amino acid) and Tr3 (1050 bp and 350 amino acid) were deposited into the GenBank database that received accession numbers of ON169974, ON169976 and ON169975, respectively. The full-length gene cassette was synthesized and cloned into pDG148 expression vector between *Hin*dIII and *Sal*I restriction sites making pDG148-hasA.

In order to design the truncated forms, the online software of TMHMM (http://www.cbs.dtu.dk/services/TMHMM/) was used for prediction of SeHAS topology. The sequence encoding Tr4 (corresponding to M_1_–H_376_ residues, without TMD5) was amplified by Tr3Tr4-F and Tr4-R primers (Table S1, Fig. S2 and Fig.S3) using pDG148-hasA as template. The coding sequence for Tr3 (corresponding to M_1_–D_350_ residues, without both TMD4 and TMD5) was also amplified using Tr3Tr4-F and Tr3-R primers (Additional file 1: Table S1, Additional file 1: Figs. S2 and S4) and pDG148-hasA as template. The *Tr4* and *Tr3* were cloned between *Hin*dIII and *Sal*I restriction sites of pDG148 plasmid resulting in pDG148-Tr4 and pDG148-Tr3 (Additional file 1: Fig. S3 and Fig.S4), respectively. The recombinant plasmids pDG148-hasA, pDG148-Tr4 and pDG148-Tr3 were then transformed into *E. coli* Top10 as propagation host via heat-shock transformation method; then after plasmids extraction and confirmation, the plasmids were separately transformed into *B. subtilis* 168 strains by the method of Anagnostopoulos and Spizizen (Anagnostopoulos and Spizizen 1961). To confirm the recombinant strains, DNA sequencing and colony PCR was performed using specific primers of SeHAS (Additional file 1: Table S1) Moreover, the expression of SeHAS and truncated forms of Tr4, Tr3 by RBSFA, RBSTr4 and RBSTr4, respectively were confirmed by the Western blotting using HRP-conjugated anti-His antibody (Sigma-Aldrich, USA) and DAB substrate (Sigma-Aldrich, USA) according to standard protocol (Ausubel 1988).

### Production and purification of HA

All recombinant strains were cultured in 5 ml of LB medium for 16–18 h at 37ºC, 200 rpm as seed cultures. The seed cultures were inoculated into 50 ml LB medium. The expression of SeHAS, Tr4 and Tr3 was induced by IPTG (final concentration of 0.6 mM) at OD_600nm_ ~ 0.5, then the HA titer and production rate were determined during 40 h fermentation in all cases. The protein impurities were removed by adding TCA 10% w/v (Tri chloroacetic acid) to culture supernatants and centrifugation at 12,000 rpm /20 min/4 °C. The supernatants was neutralized using NaOH 0.5 M then by addition of 0.5 M NaCl, the sodium hyaluronate (HA salt) was formed. The HA salt was precipitated by adding two volumes of ethanol followed by incubation at 4 °C for 18 h. The HA salt was collected by centrifugation at 12,000 rpm /20 min/4 °C and then re-suspended in distilled water. In the following, one volume of 0.1 M acetate buffer and acetone was added for removal of nucleic acid impurities and stored at − 20 °C for 2 h, eventually the HA salt was harvested by centrifugation at 12,000 rpm/20 min/4 °C and the pellet was washed with two volumes of absolute ethanol and dissolved in distilled water and stored at − 20 °C. The nucleic acid and protein impurities were determined using a UV–Vis Spectrophotometer (Thermo Fisher, USA). To investigate the effect of the purification process on HA Mw, the purification step was also performed on a control HA (Mw 880 kDa, Blomage Biotechnology Corp.)

## HA characterization

### Quantification of HA

HA titer was determined by carbazole assay with some modifications (Bitter 1962; Cesaretti et al. 2003). Briefly, a serial dilution of standard (D-glucuronic acid, Sigma) and sample solutions (50 µl) was placed in a 96-well microplate. Then 200 µl sodium tetraborate solution (0.025 M in saturated sulfuric acid) was added and mixed gently. The microplate was heated for 20 min at 80 °C. After cooling at room temperature, 50 µl carbazole (0.125% in absolute ethanol) was added to each well and mixed. After heating at 80 °C for 20 min in an oven and cooling at room temperature for 15 min, the microplate was read in a microplate reader (Epoch, BioTek, USA) at a wavelength of 550 nm. The significance of HA titer difference among strains was statistically determined using GraphPad Prism software (one-way ANOVA test, p-value < 0.05).

### FTIR spectroscopy

The identification of purified HAs was performed by FTIR analysis. For this purpose, the samples and control HA (Mw 880 kDa, Blomage Biotechnology Corp.) were dried and analyzed by spectroscopy apparatus (Thermo, USA,) at the range of 500–4000 cm^−1^.

### HA Mw determination

The Mw of purified HA from RBSFA, RBSTr4 and RBSTr3 were analyzed via agarose gel electrophoresis based on the method of Cowman et al. with some modifications (Cowman et al. 2011). Briefly, agarose gel (1% w/v) was prepared in TAE buffer (Tris-based 40 mM, Acetic acid 20 mM, EDTA 1 mM, pH 8.3) then HA samples and low Mw HA ladder (Echelon Biosciences Inc., USA) were mixed with glycerol 30% and loaded. Electrophoresis was performed at room temperature at a constant voltage of 100 V for 60 min. The gel was first fixed in 30% ethanol for 60 min and then stained by Stains-All^®^ 0.005% (1:1 of ethanol–water) for 18 h under a light protection condition. The Gel was destined by exposing the gel to the light.

## Results

### Design and construction of full-length and truncated forms of *hasA* gene

**Fig. 1:**
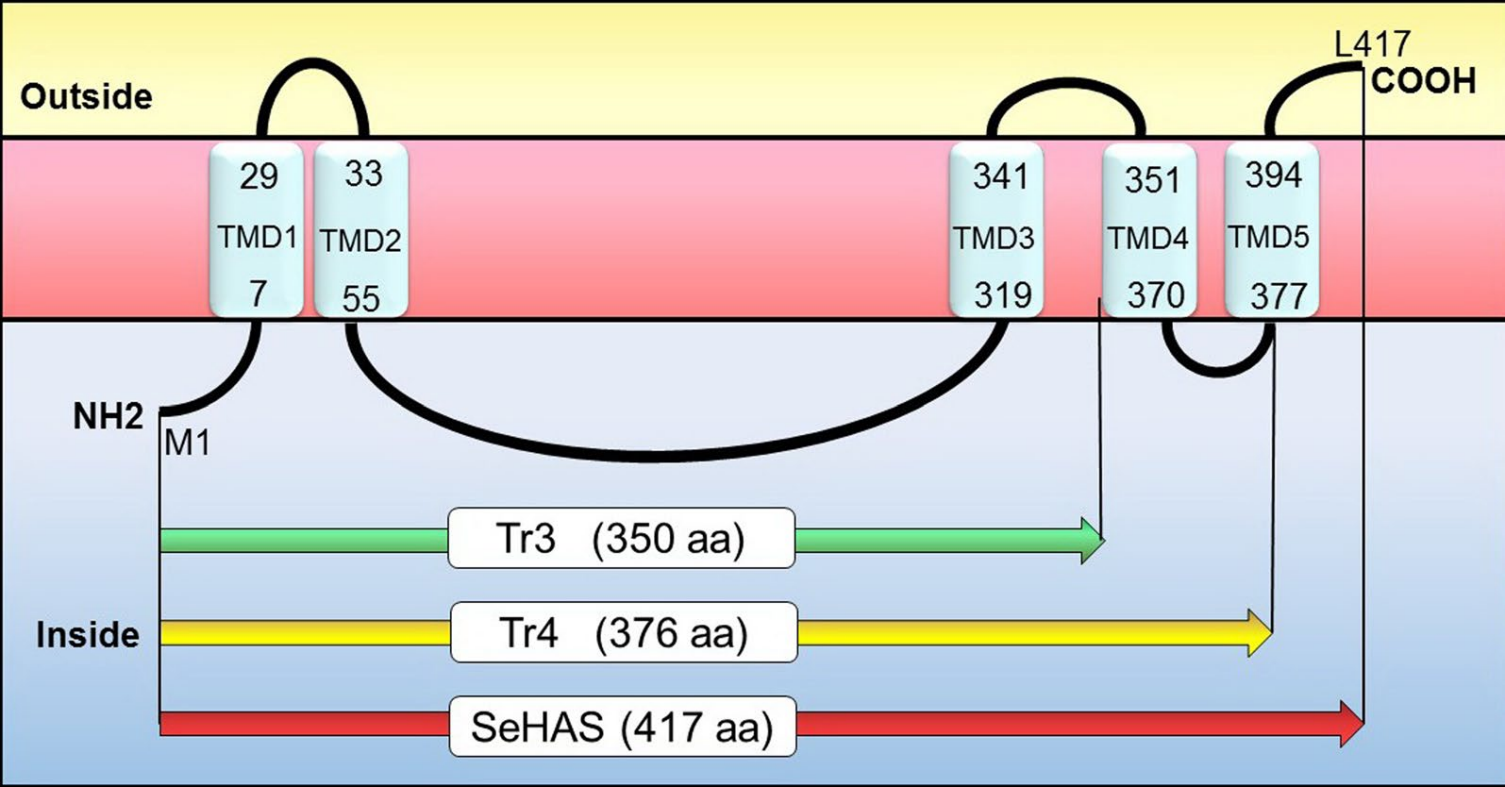
Topological model of seHAS in the membrane. The numbers within each TMD indicates its starting and ending residues

The topology prediction of SeHAS (417 residues) indicated five TMDs at positions 7–28, 33–54, 319–341, 351–370 and 377–395 residues (Fig. 1, Table2). The N-terminus (1–6aa) and C-terminus (396-417aa) regions are located inside and outside the cell, respectively (Fig. 1). The catalytic site is an intra-cellular domain located between TMD2 and TMD3 (55–318aa). The shortest α-helical domain which theoretically spans a membrane lipid bilayer is around 20 residues (Aaltonen and Silow 2008; Heldermon et al. 2001). In SeHAS, the consensus helices were between 19 and 24 amino acids in length (Fig. 1, Table 2).

**Table 2:** Topology prediction of Predicted transmembrane helices of seHAS

TMDs	Length (aa)	Average starting point	Average end point	Sequence
TMD1	23	7	29	LITVVAFSIFWVLLIYVNVYLFG
TMD2	23	33	55	SLSIYGFLLIAYLLVKMSLSFFYKK
TMD3	23	319	341	FVALWTILEVSMFMMLVYSVVDF
TMD4	20	351	370	WLRVLAFLVIIFIVALCRNI
TMD5	18	377	394	PLSFLLSPFYGVLHLFVL

According to the predicted topology of SeHAS, the Tr4 and Tr3 truncated constructs were designed in which Tr4 without TMD5 (41 amino acids deletion at c- C-terminus) and contains 1–376aa and truncated form of Tr3 without TMD4 and TMD5 consists of 1–350 aa (deletion of 67 amino acids at C-terminus) (Fig. 1).

**Fig. 2:**
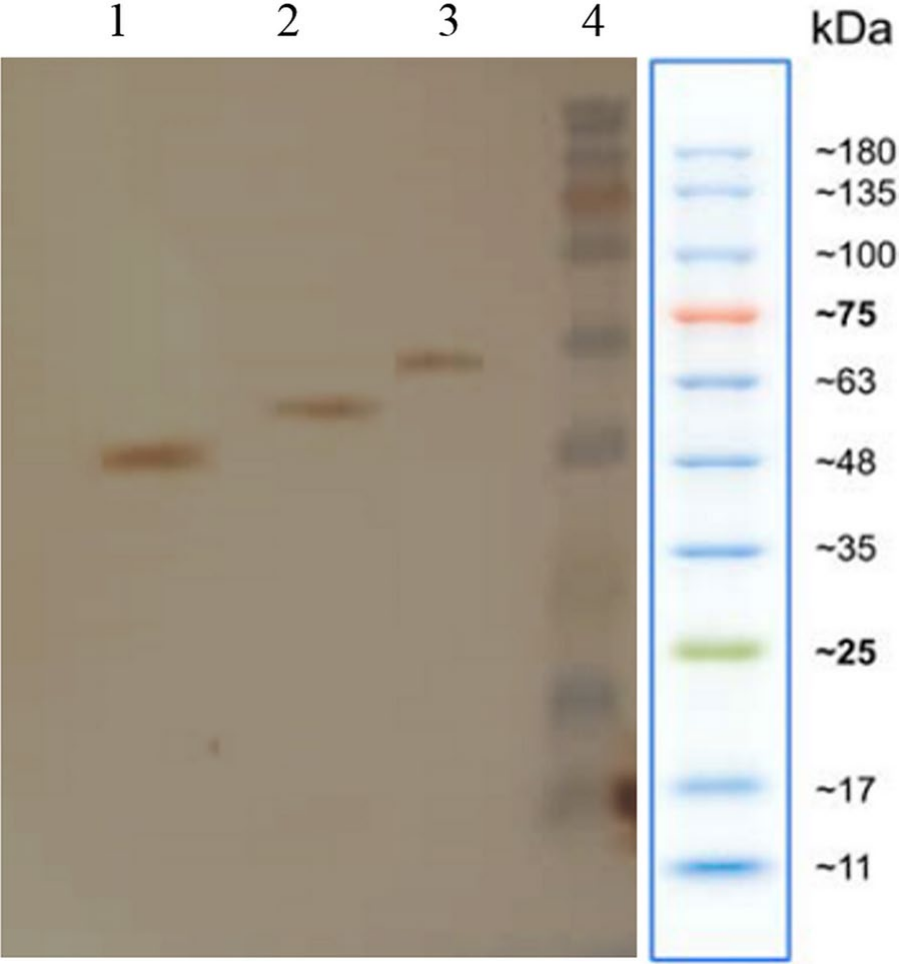
Western blot analysis of full-length and truncated forms of seHAS expressed in recombinant *Bacillus* strains (Lane 1: Tr3, Lane 2: Tr4, Lane 3: seHAS, and Lane 4: Protein ladder)

The pDG148-hasA, pDG148-Tr4 and pDG148-Tr3 were transformed into *B. subtilis* resulted in respectively recombinant RBSFA, RBSTr4 and RBSTr3 strains which were confirmed by PCR (Additional file 1: Fig.S5). The expression of SeHAS, Tr4 and Tr3 could have not been detected by SDS-PAGE analysis and Coomassie brilliant blue staining which might be due to the intrinsic hydrophobicity of these membrane proteins that reduce the efficiency of membrane protein extraction (data not shown). However, the presence of SeHAS, Tr4 and Tr3 enzymes was confirmed using western blot indicating the His-tagged protein bands at 48, 43 and 40 kDa, respectively (Fig. 2).

### Production, purification and titer determination

**Fig. 3:**
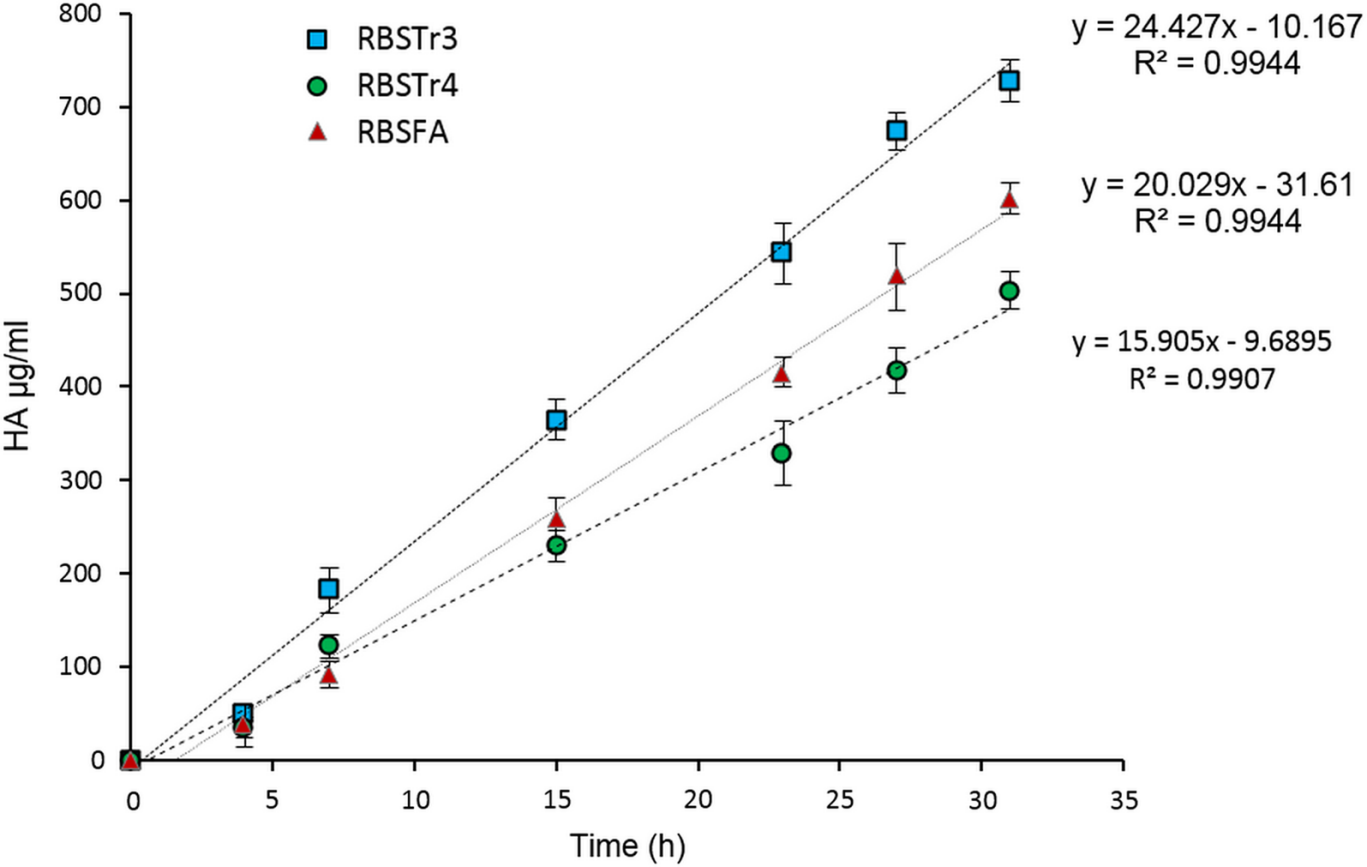
Production rate of HA by RBSFA, RBSTr4 and RBSTr3. HA production in all strains was linear until 31 h with a production rate of 15.9, 20, and 24.4 mg/L.h^−1^ for RBSFA, RBSTr4, and RBSTr3 strains, respectively

The bacterial growth and HA production by recombinant strains were monitored within 40 h (Additional file 1: Fig. S6). The growth pattern of RBSFA, RBSTr4 and RBSTr3 indicated that the maximum bacterial growth would be occurred 27 h after inoculation when optical density reached 4.46, 4.23 and 4.13 at 600 nm, respectively. Determination of HA titers indicated that the maximum HA production for recombinant strains was achieved 31 h after induction. The HA titers were 602.4 ± 16.6, 503.3 ± 19.4 and 728.3 ± 22.9 mg/L in the case of RBSFA, RBSTr4 and RBSTr3 strains, respectively. In addition, the production rate of HA by RBSFA, RBSTr4 and RBSTr3 was 20.02, 15.90, and 24.42 mg/L.h^−1^, respectively (Fig. 3 and Table 3). Statistical analysis of HA production showed that the difference in production rate among recombinant strains was significant (p-value < 0.00001).

**Table 3:** HA titer, Mw and production rate of recombinant *B. subtilis* strains

Strains	HA (mg/L)	HA MW (kDa)	HA production rate (mg/L.h^−1^)
RBSFA	602.4 ± 16.6 (100*)	55–60	20.02
RBSTr4	503.3 ± 19.4 (83*)	55–60	15.90
RBSTr3	728.3 ± 22.9 (121*)	55–60	24.42

* Relative percentage of HA titer among recombinant strains

Determination of DNA and protein impurities showed that these impurities were in the range of acceptance limit according to the HA monograph of European Pharmacopoeia 10 (Additional file 1: Table S2). Using HA control during the purification process, demonstrated that the purification steps did not have deleterious effects on HA Mw (data not shown).

### FTIR spectroscopy

**Fig. 4:**
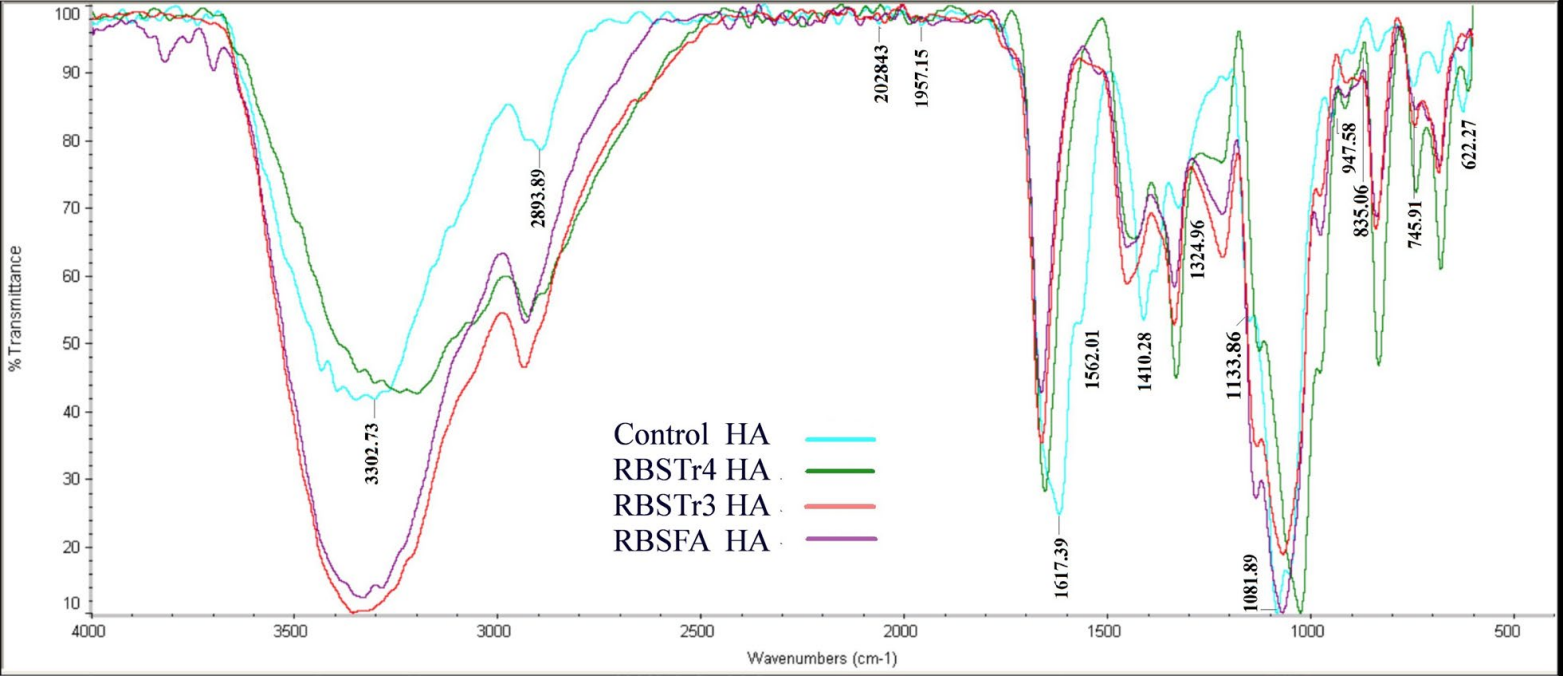
FTIR characterization of purified HAs from RBSFA, RBSTr4, and RBSTr3 strains along with standard HA

The structural identity of purified HAs was investigated by FTIR and compared to that of the control HA spectrum. The spectra analysis elucidated that there is no obvious difference between FTIR spectra of control HA and purified HA from different strains (Fig. 4). A strong absorption band was observed at 3302 cm^−1^, which indicates OH and NH bonds. The absorption at 2893 cm^−1^ was related to CH symmetrical and CH2 asymmetrical stretching. The bands at positions 1617 cm^−1^, 1562 cm^−1^, and 1324 cm^−1^ can be for amide I, II, and III. The absorption bands at 1081 cm^−1^ and 1133 cm^−1^ are typical for carbohydrates and the band at 1410 cm^−1^ is assigned to symmetric C-O stretching vibrations (Chen et al. 2019; Gilli et al. 1994).

### Determination of HA Mw

**Fig. 5:**
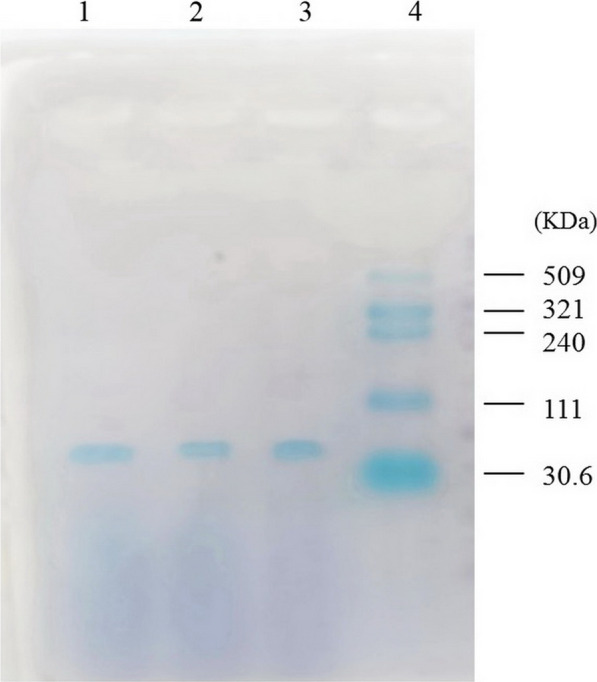
Gel Analysis of obtained HA biopolymer from RBSFA, RBSTr4, and RBSTr3 using Stains-All® method. (Lane 1: HA produced by RBSTr3, Lane 2: HA produced by RBSTr4, Lane 3: HA produced by RBSFA, and lane 4: HA ladder)

The Mw determination of purified HAs from RBSFA, RBSTr4 and RBSTr3 strains by agarose gel electrophoresis and Stains-All® staining showed the formation of a linear and non-elongated band which indicates the production of HA in all recombinant strains (Fig. 5). Estimation of the Mw was performed by gel analyzer software (Gel Analyzer version 19.1) (Additional file 1: Fig.S7). The calculations revealed an HA Mw range of ~ 55–60 KDa for all recombinant strains.

## Discussion

For HA production, the development and application of GRAS engineered strains have always been considered as an alternative solution to eliminate or reduce the natural sources related impurities. Moreover, heterologous GRAS strains would provide the tools for increasing productivity through control of fermentation conditions and overexpression of enzymes involved in the biosynthesis pathway (Chahuki et al. 2019). Since the biosynthesis pathways of HA precursors (GlcNAc and GlcA) exist in *B. subtilis*, the only requisite enzyme for HA production would be HAS enzyme (Westbrook et al. 2018b). There are several studies on cloning of *has* gene derived from *Streptococcus* strains into *B. subtilis* (Westbrook et al. 2016, 2018a, 2018b; Widner et al. 2005), however using truncated forms has not been reported before.

To find out the role function of C-terminus TMDs (TMD4 and TMD5) of SeHAS on its in vivo biological activity, in the present study the gene encoding of full and novel truncated forms (Tr4 and Tr3) of SeHAS were cloned into *B. subtilis*. The biological activity parameters including HA titer, production rate and Mw were determined and compared among recombinant strains.

The HA production measurement revealed that all recombinant strains produce HA, suggesting that the removal of TMD5 and TMD4 regions did not impair the HA production and secretion process (Table 3, Additional file 1: Fig.S6). Prior studies on the C-terminus function of SeHAS in HA productivity and Mw through amino acid deletion or substitutions have identified several hot spots that affect the in vitro SeHAS activity (Baggenstoss et al. 2017; Yang et al. 2017). The incremental or decreasing effects on HA titer has been attributed to altering the substrates and/or HA-binding affinity or loss of SeHAS the intrinsic activity (Yang et al. 2017).

The comparison of HA titer showed that RBSTr3 surprisingly produced 121% and 137% more HA than that of RBSFA and RBSTr4 strains, respectively (Table 3). It mea ns the shorter form of SeHAS (Tr3) has a higher production rate than that of intact enzyme or longer truncated form (Tr4). This could be explained by the less spatial interference of the last intracellular region (residues 370–377) with the active site of the enzyme (intracellular domain 55–319aa) and structural differences and fluctuation of enzyme function. However, more studies are needed to clarify the exact mechanism of the observed phenomenon.

Despite the HA titer and product rate variations, the HA Mw in all recombinant strains remained approximately the same (Fig. 5, Table 3) which is according to the previous report (Yang et al. 2017). These findings are strongly supported by the hypothesis that SeHAS uses two discrete functions to control HA production rate and HA Mw (Weigel and Baggenstoss 2012). In prior studies, amino acids deletion did not ultimately exceed than 20 residues at C-terminus (Baggenstoss et al. 2017; Yang et al. 2017), while here we showed even deletion of 41 (Tr4) and 67 (Tr3) amino acid residues could not affect the size of HA chain and Mw (Table 3, Fig. 5) and expanded the region that is not involved in the control of HA Mw. The HA titer by recombinant *B.subtilis* strains have been reported to vary from 460 to 1000 mg/L (Chien and Lee 2007; Jin et al. 2016). The highest titer of 728 mg/L was obtained by RBSTr3 strain (Table 3), which is comparable or higher than similar studies (Chauhan et al. 2014; Cheng et al. 2016; Chien and Lee 2007; Hmar et al. 2014; Jeong et al. 2014; Yu and Stephanopoulos 2008).

As shown in previous studies, it is logical that the co-expression of *has*A along with those enzymes that are directly involved in precursors biosynthesis such as *has* B (UDP-glucose dehydrogenase), *has* D (pyrophosphorylase) and *kfi*D (coding gene of UDP-glucose 6-dehydrogenase) would enhance the HA titer and productivity (Chauhan et al. 2014; Chien and Lee 2007; Mao et al. 2009; Yu and Stephanopoulos 2008). Therefore, the developed recombinant strains in the current study would provide a platform for the co-expression of effective genes to increase production.

It was demonstrated that HA production in different host strains has a direct correlation with bacterial growth (Shah et al. 2013; Yu and Stephanopoulos 2008). This means that as bacterial growth and HA production would be occurred simultaneously then fall down relatively just after decreasing the bacterial growth (Additional file 1: Fig. S6). This is probably due to reduced carbon and nitrogen resources and limited production of HA precursors within the cell (Shah et al. 2013).

The Mw analysis by gel electrophoresis demonstrated that all recombinant strains produced the same low Mw of HA 50–60 kDa (Table 3, Fig. 5 and Additional file 1: Fig. S7). These results are in agreement with the previous reports on recombinant *B.subtilis* that showed the HA Mw can vary from lower than 300 kDa to 4.5 MDa (Westbrook et al. 2018a, 2018b; Yang et al. 2017) The control mechanism of HA Mw is not clear, due to many intrinsic factors such as HAS enzyme structure and micro-environment, substrate availability, host background and culture condition(Gunasekaran et al. 2020; Westbrook et al. 2016). It was shown that the concentration of precursor N-GluNAc, which mediates cell wall structure too, strongly affects the Mw of HA products (Chen et al. 2009; Jeong et al. 2014). Therefore the symmetric cell growth and HA Mw and production rate require precision control of precursor concentration (Angeles and Scheffers 2021).

Although a high production rate is desirable, already control of Mw and polydispersity remain important concerns. It should be noted that HA production in recombinant microorganisms was mostly polydisperse, an important characteristic which affects its commercial value and applications (Boeriu et al. 2013). In this study, it seems that the produced HA has limited polydispersity due to its linearity and lack of elongation on the agarose gel (Fig. 5, Additional file 1: Fig. S7). The production of low-Mw HA due to modulating many important processes including morphogenesis, migration, chondrogenesis, inflammation, tumorigenesis and apoptosis (Chahuki et al. 2019) would be completely valuable. In conclusion, this study highlights the effect of C-terminus TMD deletions on in vivo activity of SeHAS and showed that the developed strains are able to produce low Mw HA at a comparable production rate and titer.

## Supplementary Information

**Additional file 1: Table S1. **Primers used in this study. **Table S2. **Nucleic acid and protein impurities in purified HA samples. **Figure S1. **The full length *hasA* expression cassette. **Figure S2**. PCR products gel electrophoresis of full-length and truncated form of *has *A. **Figure S3. **The truncated form of *Tr4* expression cassette. **Figure S4. **The truncated Tr3 expression cassette. **Figure S5. **Gel electrophoresis of colony PCR on recombinant strains. **Figure S6.** The cell growth and HA production plots. **Figure S7. **The data of GelAnalyzer software for determination of purified HA Mw

## Data Availability

The data used to support the results of this study is embedded within this article and its supporting file.

## References

[CR1] Aaltonen EK, Silow M (2008). Transmembrane topology of the Acr3 family arsenite transporter from *Bacillus subtilis*. Biochim Biophys Acta Biomembr.

[CR2] Agarwal G, Krishnan K, Prasad SB, Bhaduri A, Jayaraman G (2019). Biosynthesis of Hyaluronic acid polymer: dissecting the role of sub structural elements of hyaluronan synthase. Sci Rep.

[CR3] Anagnostopoulos C, Spizizen J (1961). Requirements for transformation in *Bacillus subtilis*. J Bacteriol.

[CR4] Angeles DM, Scheffers D-J (2021). The cell wall of *Bacillus subtilis*. Curr Issues Mol Biol.

[CR101] Ausubel FM (1988). Current protocols in molecular biology.

[CR5] Baggenstoss BA, Harris EN, Washburn JL, Medina AP, Nguyen L, Weigel PH (2017). Hyaluronan synthase control of synthesis rate and hyaluronan product size are independent functions differentially affected by mutations in a conserved tandem B-X7-B motif. Glycobiology.

[CR6] Bitter T (1962). A modified uronic acid carbazole reaction. Anal Biochem.

[CR7] Boeriu CG, Springer J, Kooy FK, van den Broek LA, Eggink G (2013). Production methods for hyaluronan. Int J Carbohydr.

[CR8] Cesaretti M, Luppi E, Maccari F, Volpi N (2003). A 96-well assay for uronic acid carbazole reaction. Carbohydr Polym.

[CR9] Chahuki FF, Aminzadeh S, Jafarian V, Tabandeh F, Khodabandeh M (2019). Hyaluronic acid production enhancement via genetically modification and culture medium optimization in *Lactobacillus acidophilus*. Int J Biol Macromol.

[CR10] Chauhan AS, Badle SS, Ramachandran K, Jayaraman G (2014). The P170 expression system enhances hyaluronan molecular weight and production in metabolically-engineered *Lactococcus lactis*. Biochem Eng J.

[CR11] Chen WY, Marcellin E, Hung J, Nielsen LK (2009). Hyaluronan molecular weight is controlled by UDP-N-acetylglucosamine concentration in *Streptococcus zooepidemicus*. J Biol Chem.

[CR12] Chen H, Qin J, Hu Y (2019). Efficient degradation of high-molecular-weight hyaluronic acid by a combination of ultrasound, hydrogen peroxide, and copper ion. Molecules.

[CR13] Cheng F, Gong Q, Yu H, Stephanopoulos G (2016). High-titer biosynthesis of hyaluronic acid by recombinant *Corynebacterium glutamicum*. Biotechnol J.

[CR14] Chien LJ, Lee CK (2007). Enhanced hyaluronic acid production in *Bacillus subtilis* by coexpressing bacterial hemoglobin. Biotechnol.

[CR15] Cowman MK, Chen CC, Pandya M, Yuan H, Ramkishun D, LoBello J, Bhilocha S, Russell-Puleri S, Skendaj E, Mijovic J (2011). Improved agarose gel electrophoresis method and molecular mass calculation for high molecular mass hyaluronan. Anal Biochem.

[CR16] Dovedytis M, Liu ZJ, Bartlett S (2020). Hyaluronic acid and its biomedical applications: a review. Eng Regen.

[CR17] Gilli R, Kacuráková M, Mathlouthi M, Navarini L, Paoletti S (1994). FTIR studies of sodium hyaluronate and its oligomers in the amorphous solid phase and in aqueous solution. Carbohydr Res.

[CR18] Greenlee H, DuPont-Reyes MJ, Balneaves LG, Carlson LE, Cohen MR, Deng G, Johnson JA, Mumber M, Seely D, Zick SM (2017). Clinical practice guidelines on the evidence-based use of integrative therapies during and after breast cancer treatment. CA Cancer J Clin.

[CR19] Gunasekaran V, Gowdhaman D, Ponnusami V (2020). Role of membrane proteins in bacterial synthesis of hyaluronic acid and their potential in industrial production. Int J Biol Macromol.

[CR20] Harwood CR (1992). *Bacillus subtilis* and its relatives: molecular biological and industrial workhorses. Trends Biotechnol.

[CR21] Heldermon C, DeAngelis PL, Weigel PH (2001). Topological organization of the hyaluronan synthase from *streptococcus pyogenes*. J Biol Chem.

[CR22] Hmar RV, Prasad SB, Jayaraman G, Ramachandran KB (2014). Chromosomal integration of hyaluronic acid synthesis (has) genes enhances the molecular weight of hyaluronan produced in *Lactococcus lactis*. Biotechnol J.

[CR23] Jeong E, Shim WY, Kim JH (2014). Metabolic engineering of *Pichia pastoris* for production of hyaluronic acid with high molecular weight. J Biotechnol.

[CR24] Jin P, Kang Z, Yuan P, Du G, Chen J (2016). Production of specific-molecular-weight hyaluronan by metabolically engineered *Bacillus subtilis* 168. Metab Eng.

[CR25] Kogan G, Šoltés L, Stern R, Gemeiner P (2007). Hyaluronic acid: a natural biopolymer with a broad range of biomedical and industrial applications. Biotechnol Lett.

[CR26] Liu K, Catchmark JM (2019). Bacterial cellulose/hyaluronic acid nanocomposites production through co-culturing *Gluconacetobacter hansenii* and *Lactococcus lactis* in a two-vessel circulating system. Bioresour Technol.

[CR27] Liu L, Liu Y, Li J, Du G, Chen J (2011). Microbial production of hyaluronic acid: current state, challenges, and perspectives. Microb Cell Fact.

[CR28] Manfrão-Netto JH, Queiroz EB, de Oliveira Junqueira AC, Gomes AM, Gusmao de Morais D, Paes HC, Parachin NS (2022). Genetic strategies for improving hyaluronic acid production in recombinant bacterial culture. J Appl Microbiol.

[CR29] Mao Z, Shin H-D, Chen R (2009). A recombinant *E*. *coli* bioprocess for hyaluronan synthesis. Appl Microbiol Biotechnol.

[CR30] Rehm BH (2010). Bacterial polymers: biosynthesis, modifications and applications. Nat Rev Microbiol.

[CR31] Sambrook J, Russell DW (2006). Preparation and transformation of competent E coli using calcium chloride. Cold Spring Harbor Protocols.

[CR32] Shah MV, Badle SS, Ramachandran KB (2013). Hyaluronic acid production and molecular weight improvement by redirection of carbon flux towards its biosynthesis pathway. Biochem Eng J.

[CR33] Stragier P, Bonamy C, Karmazyn-Campelli C (1988). Processing of a sporulation sigma factor in *Bacillus subtilis*: how morphological structure could control gene expression. Cell.

[CR34] Sze JH, Brownlie JC, Love CA (2016). Biotechnological production of hyaluronic acid: a mini review. 3 Biotech.

[CR35] Waeijen-Smit K, Reynaert NL, Beijers RJ, Houben-Wilke S, Simons SO, Spruit MA, Franssen FM (2021). Alterations in plasma hyaluronic acid in patients with clinically stable COPD versus (non) smoking controls. Sci Rep.

[CR36] Weigel PH, Baggenstoss BA (2012). Hyaluronan synthase polymerizing activity and control of product size are discrete enzyme functions that can be uncoupled by mutagenesis of conserved cysteines. Glycobiology.

[CR37] Westbrook AW, Moo-Young M, Chou CP (2016). Development of a CRISPR-Cas9 tool kit for comprehensive engineering of *Bacillus subtilis*. Appl Environ Microbiol.

[CR38] Westbrook AW, Ren X, Moo-Young M, Chou CP (2018). Engineering of cell membrane to enhance heterologous production of hyaluronic acid in *Bacillus subtilis*. Biotechnol Bioeng.

[CR39] Westbrook AW, Ren X, Oh J, Moo-Young M, Chou CP (2018). Metabolic engineering to enhance heterologous production of hyaluronic acid in *Bacillus subtilis*. Metab Eng.

[CR40] Widner B, Behr R, Von Dollen S, Tang M, Heu T, Sloma A, Sternberg D, DeAngelis PL, Weigel PH, Brown S (2005). Hyaluronic acid production in *Bacillus subtilis*. Appl Environ Microbiol.

[CR41] Yang J, Cheng F, Yu H, Wang J, Guo Z, Stephanopoulos G (2017). Key role of the carboxyl terminus of hyaluronan synthase in processive synthesis and size control of hyaluronic acid polymers. Biomacromol.

[CR42] Yoshimura T, Shibata N, Hamano Y, Yamanaka K (2015). Heterologous production of hyaluronic acid in an ε-Poly-l-Lysine producer *Streptomyces Albulus*. Appl Environ Microbiol.

[CR43] Yu H, Stephanopoulos G (2008). Metabolic engineering of *Escherichia coli* for biosynthesis of hyaluronic acid. Metab Eng.

