# Supplementary material for: Hyaluronic acid production and characterization by novel Bacillus subtilis harboring truncated Hyaluronan Synthase
Source: AMB Express. 2022 Jul 12;12:88. doi: 10.1186/s13568-022-01429-3 (PMC9445140; doi:10.1186/s13568-022-01429-3)
Supplement: Supplementary file 1 — Additional file 1: Table S1. Primers used in this study. Table S2. Nucleic acid and protein impurities in purified HA samples. Figure S1. The full length hasA expression cassette. Figure S2. PCR products gel electrophoresis of full-length and truncated form of has A. Figure S3. The truncated form of Tr4 expression cassette. Figure S4. The truncated Tr3 expression cassette. Figure S5. Gel electrophoresis of colony PCR on recombinant strains. Figure S6. The cell growth and HA production plots. Figure S7. The data of GelAnalyzer software for determination of purified HA Mw [file 13568_2022_1429_MOESM1_ESM.docx]

**AMB Express**

**Hyaluronic acid production and characterization by novel *Bacillus subtilis* harboring truncated Hyaluronan Synthase**

Fatemeh Sadat Amjad Zanjani^1^, Shadi Afrasiabi^1^, Dariush Norouzian^1^, Gholamreza Ahmadian^2^, Sara Ali Hosseinzadeh^1^, Alireza Fayazi Barjin^1^, Reza Ahangari Cohan^1*^, Malihe Keramati^1*^

^1^Department of Nanobiotechnology, New Technologies Research Group, Pasteur Institute of Iran, Tehran, Iran

^2^Department of Industrial and Environmental Biotechnology, National Institute for Genetic Engineering and Biotechnology (NIGEB), Tehran, Iran

***Corresponding authors:**

Department of Nanobiotechnology, New Technologies Research Group, Pasteur Institute of Iran, Tehran, Iran, Tel: (98)2164112126, Email: Email: [cohan_r@yahoo.com](mailto:cohan_r@yahoo.com), and

[keramati.malihe@gmail.com](mailto:keramati.malihe@gmail.com), [keramatim@pasteur.ac.ir](mailto:keramatim@pasteur.ac.ir) , 0000-002-7196-8751

**Table S1.** Primers used in this study.

| Primer Name | Sequence (5ˊ→ 3ˊ) |
| --- | --- |
| Tr3tr4-F | CGTAAGCTTCCCGGGATGAGAACAC |
| Tr4-R | ATTGCTAGCGTGGTGGTGGTGGTGGTGATGTTTCAGCATA |
| Tr3-R | ATAGCTAGCGTGGTGGTGGTGGTGGTGATCAAATTCTCTAAC |

**Table S2.** Nucleic acid and protein impurities in purified HA samples.

| **Strains** | DNA impurity | Protein impurity | |
| --- | --- | --- | --- |
|  | (A_260 nm_) | (mg/L) | % |
| RBSFA | 0.022±0.004 | 0.6±0.2 | 0.06±0.2 |
| RBSTr3 | 0.019±0.007 | 0.4±0.3 | 0.04±0.3 |
| RBSTr4 | 0.021±0.006 | 0.3±0.3 | 0.03±0.3 |
| Acceptance limit according to EP (HA monograph) ^a^ | ≤0.5 | ≤1 | ≤0.1 |

^a^ European Pharmacopeia 10

**A)**

*Terminator*

**His6-tag**

***Hin*dIII**

*hasA*

***Nhe*I**

***Sal*I**

***Nhe*I**

AAGCTTATGAGAACACTTAAAAATCTGATCACGGTTGTTGCATTTAGCATCTTTTGGGTTCTGCTGATCTATGTTAATGTTTATTTATTTGGCGCAAAAGGCTCACTGTCAATCTATGGCTTTCTGCTGATCGCATATTTACTGGTTAAAATGAGCCTTAGCTTTTTTTATAAACCGTTTAAAGGAAGAGCAGGCCAATATAAAGTTGCAGCAATTATCCCGAGCTATAATGAAGATGCAGAATCATTACTTGAAACACTTAAATCAGTTCAACAACAAACGTATCCTCTGGCAGAAATCTATGTTGTTGATGATGGAAGCGCAGATGAAACAGGAATTAAACGCATCGAAGATTATGTTCGCGATACGGGGGATCTCTCTTCTAATGTTATCGTTCATCGCTCAGAAAAAAATCAAGGCAAACGCCATGCACAAGCATGGGCATTTGAACGCTCAGATGCAGATGTTTTTCTTACAGTTGATAGCGATACGTATATCTATCCGGATGCACTTGAAGAACTTCTTAAAACATTTAATGATCCGACAGTTTTTGCAGCAACGGGACATCTTAATGTTAGAAATAGACAAACGAATTTACTGACACGCCTGACGGATATTCGCTATGATAATGCATTTGGAGTTGAACGCGCAGCACAAAGCGTTACAGGCAATATCCTGGTTTGCTCAGGACCTCTGTCAGTTTATAGACGCGAAGTTGTTGTTCCTAATATCGATAAATATATTAATCAAACGTTTCTTGGGATTCCGGTTAGCATCGGCGATGATCGCTGCCTGACGAATTATGCAACGGATCTGGGCAAAACGGTTTATCAATCTACAGCAAAATGCATTACGGATGTTCCGGATAAAATGTCTACGTATCTTAAACAACAAAATAGATGGAATAAATCGTTTTTTCGCGAATCAATTATCTCAGTTAAAAAAATCATGAATAATCCGTTTGTTGCACTGTGGACGATCCTGGAAGTTAGCATGTTTATGATGCTGGTTTATAGCGTTGTTGATTTTTTTGTTGGCAATGTTAGAGAATTTGATTGGCTGCGCGTTCTGGCATTTCTGGTTATTATCTTTATCGTTGCACTGTGCCGCAATATTCATTATATGCTGAAACATCCGCTGAGCTTTCTGCTGTCACCGTTTTATGGAGTTCTTCATTTATTTGTTCTTCAACCTCTGAAACTGTATAGCCTGTTTACGATTCGCAATGCAGATTGGGGCACACGCAAAAAACTGCTTGCTAGCCATCATCATCATCATCAT**TAA**GCTAGC**TAA**GTGTGAAAAAAAGCGCAGCTGAAATAGCTGCGCTTTTTTGTGTCATAACCCTTTACAGTCAGCGCAGCTGAAATAGCTGCGCGTCGAC

**B)**

**
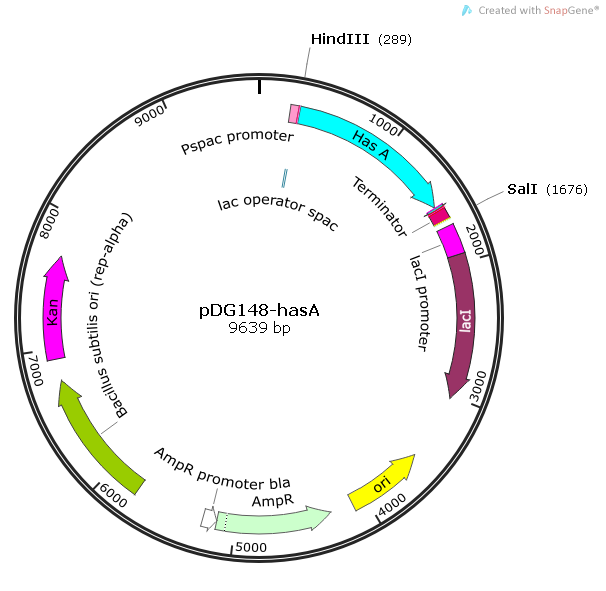
**

**Fig. S1. The full length *hasA* expression cassette.** **(A)** Construct harboring *hasA* gene along with restriction sites, termination codon and terminator region. **(B)** *E. coli-Bacillus* shuttle expression vector pDG148-hasA including full-length *hasA* gene (1251bp).

**
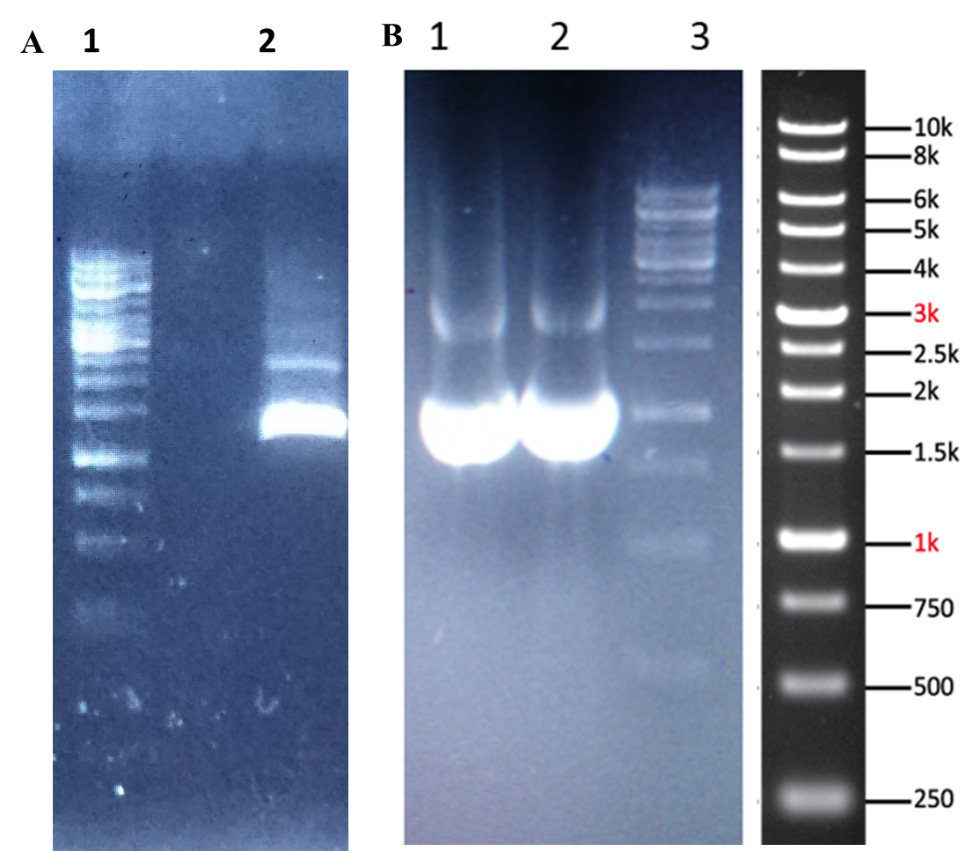
**

**Fig. S2.** **PCR products gel electrophoresis of full-length and truncated form of *has* A**. (**A)**: Lane 1: DNA marker, Lane 2: full-length *has* A (1251bp), (**B):** Lane 1 *Tr4* (1128 bp), Lane 2: *Tr3* (1050), and Lane 3: DNA marker.

**A)**

*Terminator*

**His6-tag**

***Hin*dIII**

*Tr4*

***Nhe*I**

***Sal*I**

***Nhe*I**

AAGCTTATGAGAACACTTAAAAATCTGATCACGGTTGTTGCATTTAGCATCTTTTGGGTTCTGCTGATCTATGTTAATGTTTATTTATTTGGCGCAAAAGGCTCACTGTCAATCTATGGCTTTCTGCTGATCGCATATTTACTGGTTAAAATGAGCCTTAGCTTTTTTTATAAACCGTTTAAAGGAAGAGCAGGCCAATATAAAGTTGCAGCAATTATCCCGAGCTATAATGAAGATGCAGAATCATTACTTGAAACACTTAAATCAGTTCAACAACAAACGTATCCTCTGGCAGAAATCTATGTTGTTGATGATGGAAGCGCAGATGAAACAGGAATTAAACGCATCGAAGATTATGTTCGCGATACGGGGGATCTCTCTTCTAATGTTATCGTTCATCGCTCAGAAAAAAATCAAGGCAAACGCCATGCACAAGCATGGGCATTTGAACGCTCAGATGCAGATGTTTTTCTTACAGTTGATAGCGATACGTATATCTATCCGGATGCACTTGAAGAACTTCTTAAAACATTTAATGATCCGACAGTTTTTGCAGCAACGGGACATCTTAATGTTAGAAATAGACAAACGAATTTACTGACACGCCTGACGGATATTCGCTATGATAATGCATTTGGAGTTGAACGCGCAGCACAAAGCGTTACAGGCAATATCCTGGTTTGCTCAGGACCTCTGTCAGTTTATAGACGCGAAGTTGTTGTTCCTAATATCGATAAATATATTAATCAAACGTTTCTTGGGATTCCGGTTAGCATCGGCGATGATCGCTGCCTGACGAATTATGCAACGGATCTGGGCAAAACGGTTTATCAATCTACAGCAAAATGCATTACGGATGTTCCGGATAAAATGTCTACGTATCTTAAACAACAAAATAGATGGAATAAATCGTTTTTTCGCGAATCAATTATCTCAGTTAAAAAAATCATGAATAATCCGTTTGTTGCACTGTGGACGATCCTGGAAGTTAGCATGTTTATGATGCTGGTTTATAGCGTTGTTGATTTTTTTGTTGGCAATGTTAGAGAATTTGATTGGCTGCGCGTTCTGGCATTTCTGGTTATTATCTTTATCGTTGCACTGTGCCGCAATATTCATTATATGCTGAAACATGCTAGCCATCATCATCATCATCAT**TAA**GCTAGC**TAA**GTGTGAAAAAAAGCGCAGCTGAAATAGCTGCGCTTTTTTGTGTCATAACCCTTTACAGTCAGCGCAGCTGAAATAGCTGCGCGTCGAC

**B)**

**
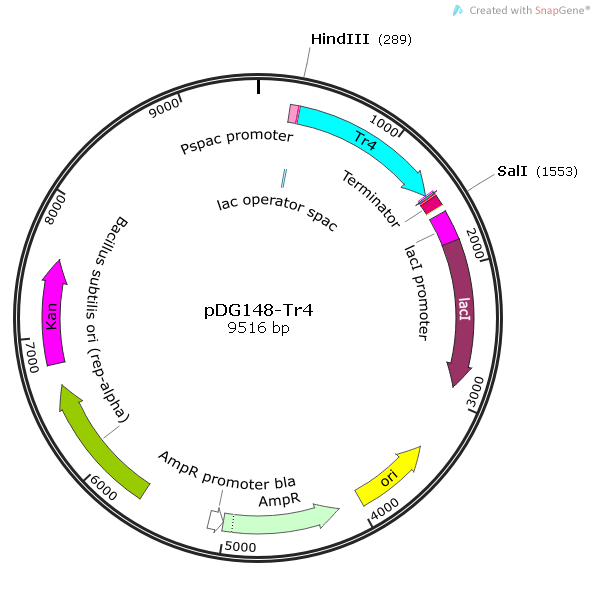
**

**Fig. S3. The truncated form of *Tr4* expression cassette.** **(A)** Construct harboring *Tr4* gene along with restriction sites, termination codon, and terminator regions. **(B)** *E. coli-Bacillus* shuttle expression vector pDG148-Tr4 including *Tr4* gene (1128bp).

**A)**

*Terminator*

**His6-tag**

***Hin*dIII**

*Tr3*

***Nhe*I**

***Sal*I**

***Nhe*I**

AAGCTTATGAGAACACTTAAAAATCTGATCACGGTTGTTGCATTTAGCATCTTTTGGGTTCTGCTGATCTATGTTAATGTTTATTTATTTGGCGCAAAAGGCTCACTGTCAATCTATGGCTTTCTGCTGATCGCATATTTACTGGTTAAAATGAGCCTTAGCTTTTTTTATAAACCGTTTAAAGGAAGAGCAGGCCAATATAAAGTTGCAGCAATTATCCCGAGCTATAATGAAGATGCAGAATCATTACTTGAAACACTTAAATCAGTTCAACAACAAACGTATCCTCTGGCAGAAATCTATGTTGTTGATGATGGAAGCGCAGATGAAACAGGAATTAAACGCATCGAAGATTATGTTCGCGATACGGGGGATCTCTCTTCTAATGTTATCGTTCATCGCTCAGAAAAAAATCAAGGCAAACGCCATGCACAAGCATGGGCATTTGAACGCTCAGATGCAGATGTTTTTCTTACAGTTGATAGCGATACGTATATCTATCCGGATGCACTTGAAGAACTTCTTAAAACATTTAATGATCCGACAGTTTTTGCAGCAACGGGACATCTTAATGTTAGAAATAGACAAACGAATTTACTGACACGCCTGACGGATATTCGCTATGATAATGCATTTGGAGTTGAACGCGCAGCACAAAGCGTTACAGGCAATATCCTGGTTTGCTCAGGACCTCTGTCAGTTTATAGACGCGAAGTTGTTGTTCCTAATATCGATAAATATATTAATCAAACGTTTCTTGGGATTCCGGTTAGCATCGGCGATGATCGCTGCCTGACGAATTATGCAACGGATCTGGGCAAAACGGTTTATCAATCTACAGCAAAATGCATTACGGATGTTCCGGATAAAATGTCTACGTATCTTAAACAACAAAATAGATGGAATAAATCGTTTTTTCGCGAATCAATTATCTCAGTTAAAAAAATCATGAATAATCCGTTTGTTGCACTGTGGACGATCCTGGAAGTTAGCATGTTTATGATGCTGGTTTATAGCGTTGTTGATTTTTTTGTTGGCAATGTTAGAGAATTTGATGCTAGCCATCATCATCATCATCAT**TAA**GCTAGC**TAA**GTGTGAAAAAAAGCGCAGCTGAAATAGCTGCGCTTTTTTGTGTCATAACCCTTTACAGTCAGCGCAGCTGAAATAGCTGCGCGTCGAC

**B)**

**
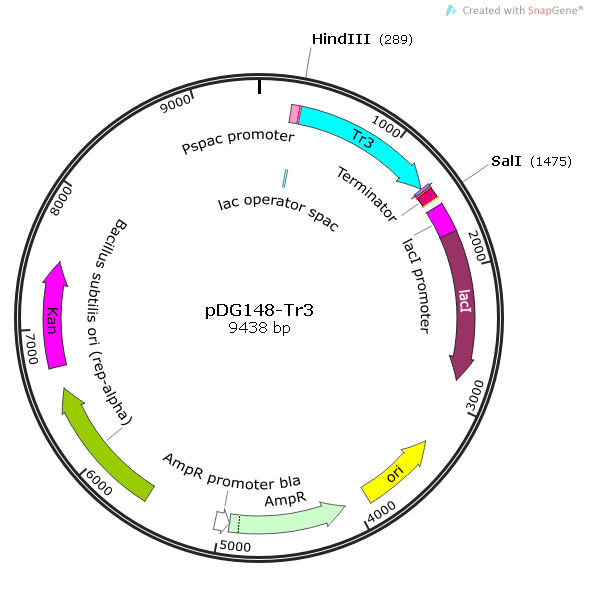
**

**Fig. S4. The truncated Tr3 expression cassette**. **(A)** Construct harboring *Tr3* gene along with cutting sites, termination codon, and terminator region. **(B)** *E. coli-Bacillus* shuttle expression vector pDG148-Tr3 including *Tr3* gene (1050 bp).


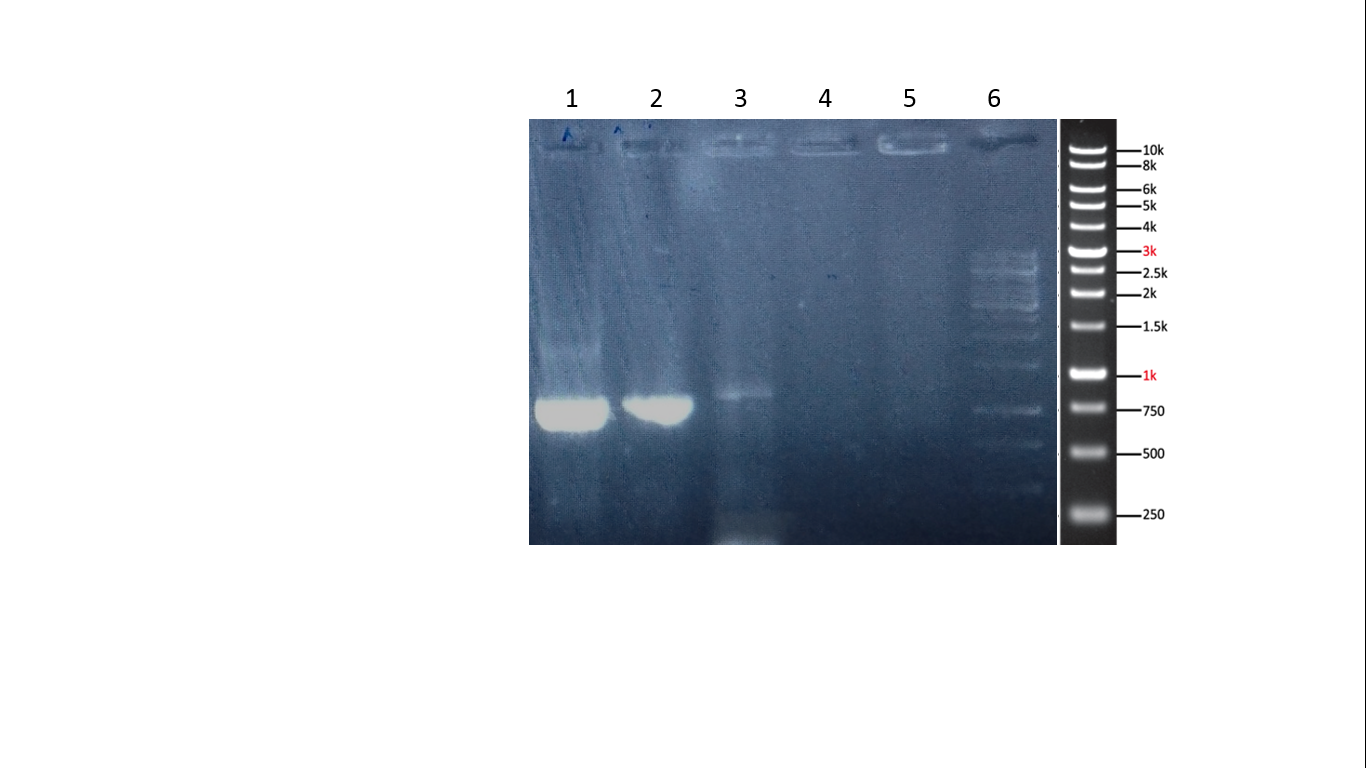


**Fig. S5. Gel electrophoresis of colony PCR on recombinant strains.** Lane 1: RBSTr3 harboring pDG148-*Tr3* vector, Lane 2: RBSTr4 harboring pDG148-*Tr4* vector, Lane 3: RBSFA harboring pDG148-hasA vector, Lanes 4-5: Negative control, and Lane 6: DNA marker. The faint band of full-length *hasA* was probably due to the small number of primary colonies taken.

**Fig. S6. The cell growth and HA production plots.** **(A)** HA production plots of RBSFA, RBSTr4, and RBSTr3 strains during from the starting point of inoculation up to 40 h. **(B)** Cell growth curves of RBSFA, RBSTr4, and RBSTr3 strains. The data are represented as Mean ± standard deviations from three independent experiments.

**(A) (B)**

**
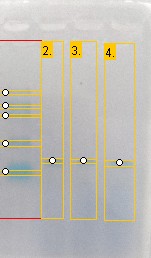

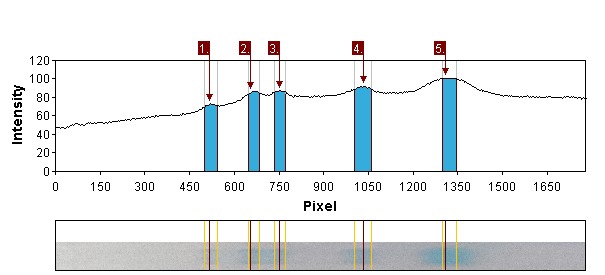
**

**(C)**

| Band Number | Relative Migration (Rf) | Molecular Weight (MW) |
| --- | --- | --- |
| 1 | 0.29 | 509 |
| 2 | 0.367 | 321 |
| 3 | 0.421 | 240 |
| 4 | 0.58 | 111 |
| 5 | 0.734 | 31 |

**(D)**

**
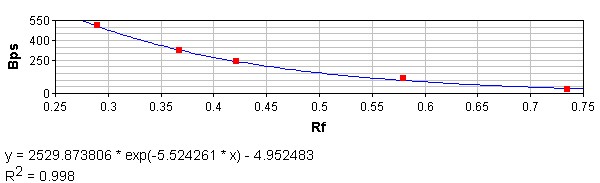
**

**Fig. S7. The data of GelAnalyzer software for determination of purified HA Mw**. Briefly, the distance of relative migration (Rf) of HA bands of ladder were measured (**A, B,** and **C**). Then, the known Mw of HA ladder (y-axis) were plotted against their relative migration (x-axis). A co-efficient of determination (R2) of 0.998 was obtained from linear equation **(D)**. Finally, the Mw of purified HA samples were calculated using the equation.
